# Limiting the Spread of Multidrug-Resistant Bacteria in Low-to-Middle-Income Countries: One Size Does Not Fit All

**DOI:** 10.3390/pathogens12010144

**Published:** 2023-01-14

**Authors:** Rindala Saliba, Jean-Ralph Zahar, Georges Dabar, Moussa Riachy, Dolla Karam-Sarkis, Rola Husni

**Affiliations:** 1 Clinical Microbiology Department, Hotel-Dieu de France University Hospital, Beirut 1100, Lebanon; 2 Laboratoire des Agents Pathogènes, Saint-Joseph University of Beirut, Beirut 1100, Lebanon; 3 Faculty of Medicine, Saint-Joseph University of Beirut, Beirut 1100, Lebanon; 4 Infection Prevention and Control Department, Avicenne University Hospital, 93000 Bobigny, France; 5 Pulmonology and Critical Care Department, Hotel-Dieu de France University Hospital, Beirut 1100, Lebanon; 6 School of Medicine, Lebanese American University, Beirut 1100, Lebanon; 7 Lebanese American University Medical Center, Rizk Hospital, Beirut 1100, Lebanon

**Keywords:** infection control, multidrug-resistant organisms, antimicrobial resistance and low-to-middle income countries

## Abstract

The spread of multidrug-resistant organisms (MDRO) is associated with additional costs as well as higher morbidity and mortality rates. Risk factors related to the spread of MDRO can be classified into four categories: bacterial, host-related, organizational, and epidemiological. Faced with the severity of the MDRO predicament and its individual and collective consequences, many scientific societies have developed recommendations to help healthcare teams control the spread of MDROs. These international recommendations include a series of control measures based on surveillance cultures and the application of barrier measures, ranging from patients’ being isolated in single rooms, to the reinforcement of hand hygiene and implementation of additional contact precautions, to the cohorting of colonized patients in a dedicated unit with or without a dedicated staff. In addition, most policies include the application of an antimicrobial stewardship program. Applying international policies to control the spread of MDROs presents several challenges, particularly in low-to-middle-income countries (LMICs). Through a review of the literature, this work evaluates the real risks of dissemination linked to MDROs and proposes an alternative policy that caters to the means of LMICs. Indeed, sufficient evidence exists to support the theory that high compliance with hand hygiene and antimicrobial stewardship reduces the risk of MDRO transmission. LMICs would therefore be better off adopting such low-cost policies without necessarily having to implement costly isolation protocols or impose additional contact precautions.

## 1. General Introduction: The Threat of MDROs and Necessity of Limiting Their Spread

Antimicrobial resistance is a major global health problem. The spread of multidrug-resistant organisms (MDRO) is associated with additional costs as well as higher morbidity and mortality rates. The World Health Organization (WHO) has classified the fight against the spread of MDROs as a “critical” priority [1].

Colonization with an MDRO, whether community-acquired or nosocomial, exposes a patient to a higher risk of infection. In turn, this can lead to inadequate therapeutic remedies or even therapeutic failure, and by extension, to increased mortality. In the United States, more than 2.8 million antibiotic-resistant infections occur each year, resulting in 35,000 deaths [2].

Over the past two decades, resistance mechanisms within commensal bacteria frequently responsible for infections have emerged and become widely spread. One of the most threatening particularities of certain resistance mechanisms is their transferable nature, considering that they are carried by mobile genetic elements such as integrons, transposons, and plasmids [3]. Initially, extended-spectrum β-lactamases (ESBL) represented a resistance mechanism that inactivates all β-lactams except carbapenems, although there remained many possible therapeutic options. Subsequent years have witnessed the unfolding and spreading of carbapenemase-producing *Enterobacterales* (CPE) and vancomycin-resistant enterococci (VRE), which pose real therapeutic problems insofar as, at least until very recently, there were few or no therapeutic options [4,5,6,7]. Following the introduction of carbapenems in therapy during the early 1980s, the description of resistance by enzymatic hydrolysis in *Enterobacterales* was not established until 2001, when the first strain of *Klebsiella pneumoniae* harboring a KPC-carbapenemase was published. [8] Between 2006 and 2007, a study including 463 American hospitals already showed the prevalence of KPC-producing strains of *Klebsiella pneumoniae* to be 10%. After 2005, outbreaks were described in Greece, Israel, and Italy [3]. This rapid success was based on the diffusion of the *blaKPC* resistance gene in a predominant clone, clone ST-258, which was found in different outbreaks. Subsequently, other genes conferring resistance to carbapenems have been found in outbreaks across the planet [3]. To date, many CPE endemic foci persist around the Mediterranean Sea and in southern Europe. Certain countries report a very high prevalence of resistance to carbapenems, including Italy, Greece, Turkey, Israel, Cyprus, and Malta [9,10,11,12].

Enterococci are microorganisms frequently found in healthcare-associated infections. From the first description of a strain of *Enterococcus faecium* resistant to vancomycin in 1988, VRE has spread across the world to achieve very high resistance rates in certain countries. The emergence of this resistance has been attributed to selection caused by avoparcin, a glycopeptide used as a growth factor in veterinary medicine [13]. Data from the European antimicrobial resistance surveillance network shows a high prevalence, exceeding 20%, of VRE in Cyprus, Greece, Ireland, Romania, Germany, the United Kingdom, and Italy, compared to less than 1% in France and Belgium [14]. In the United States, the vancomycin resistance rate in *Enterococcus faecium* bacteremia increased from 57.1% in 2000 to 80.7% in 2010 [15].

The proliferation of MDROs in hospital and community settings, along with the ensuing increase in morbidity, mortality, and care costs and the fear of a therapeutic impasse, justify the interest in the fight against acquisition and transmission of MDROs. This is a veridical challenge for clinicians, microbiologists, and healthcare authorities [16], as the transmission dynamics include both the colonized or infected patients (reservoir) and the healthcare professionals (direct vector) and environment (indirect vector) in which they operate [17,18].

Hospital-acquired infections worldwide are mostly due to the top five bacterial species with relevant intrinsic resistance and extensive capacity to acquire multi-drug resistance. These bacterial species are *Enterococcus faecium*, *Staphylococcus aureus*, *Klebsiella pneumoniae*, *Acinetobacter baumannii*, *Pseudomonas aeruginosa*, *Enterobacter* spp., and *Escherichia coli* (ESKAPE-E) [19,20]. Even though comprehensive data are scarce, previous studies suggest that low-to-middle-income countries (LMICs) are likely to be most affected by the declining effectiveness of antibiotics. LMICs’ higher exposure is by dint of pre-existing developmental and economical challenges and likely deficiencies in the healthcare system. [21,22]. A systematic review by Allengranzi et al. conducted in 2011 showed that the burden of hospital-acquired infections is higher in LMICs in comparison with high-income countries (HICs) [22]. More recently, in their 2022 systematic review and meta-analysis, Ayobami et al. showed that pooled resistance proportions of hospital-acquired ESKAPE-E infections to critical antibiotics in low- and lower–middle-income are generally high (range: 16.6–85.5%). Higher resistance to antibiotics exacerbates the broader socio-economic challenges that exist in resource-stricken regions, potentially further constraining their healthcare systems and exponentially impacting the livelihoods of the affected communities [23]. Similarly, antimicrobial resistance in intensive care unit-acquired infections is higher in LMICs than in HICs, as per Saharman et al.’s 2021 scoping review. The pathogen distribution is different as well [24].

In order to curb the transmission of MDROs, international studies have defined several courses of intervention which have become the basis of international infection-prevention policies in hospital settings. These behavioral recommendations rely on screening, isolation, and cohorting strategies for colonized patients. They have indeed been proven widely effective in the international literature and by empirical evidence [25,26]. However, the same cannot be postulated for LMICs. Applying international policies to control the spread of MDROs is an inconceivable task when resources are strapped, such in the case of LMICs. While the overarching strategies to combat MDROs are well described at the global level, the varying regional MDRO patterns in LMICs suggest the need for priorities to be redirected. Doing this can aid in understanding the MDRO dynamics at regional and local levels, thereby establishing evidence to tailor sustainable and realistically achievable solutions [23].

Easy movement between borders, geopolitical tensions, and population exchange between LMICs and HICs are at the basis of current scientific and epidemiological concerns. Thus, controlling the spread of MDROs in HICs alone without finding remedies achievable in LMICs is insufficient to contain the global spread of resistance and prevent future pandemics. Having identified the impracticability of implementing the proposed international measures in LMIC settings, this review proposes an alternative policy adapted to the means of LMICs that can ensure better control over the spread of MDROs. Among all known MDROs to date, in this review we primarily discuss CPE and VRE, as these are considered a community and hospital-acquired burden worldwide. First, in the following chapters, we review the literature in order to evaluate the real risks of dissemination linked to MDROs, CPE and VRE in particular, and provide a holistic understanding of the conundrum at hand.

## 2. Transmission Pathways of MDROs in Hospitals and at the Community Level

In order for MDROs to be transmitted from a colonized or infected patient to another patient, there are several prerequisites. First, a carrier must disseminate the MDRO through (1) a vector, such as a healthcare worker, and (2) direct contact with the contaminated environment, such as via colonized skin or excretions of contaminated body fluids. In the environment, the MDRO must survive to be transferred, either directly to other patients or indirectly through healthcare workers.

In addition, the potential sources of spread of MDROs in the community are multifold. For instance, several reservoirs have been identified as being at risk of MDRO introduction into the community. A patient who has acquired an MDRO in the hospital can contaminate their own environment at home or at work. A trip to an endemic area can be another source of MDRO colonization. Contaminated food (meat or vegetables) can be a potential source. Finally, MDROs can be disseminated in the natural environment through fecal contamination [27].

Risk factors related to the spread of MDROs in the hospital setting can be classified into four categories: (1) bacterial, including different bacterial species, successful clones, and their ability to survive in the environment; (2) host-related, including gut microbiota composition as well as antibiotic and non-antibiotic selection pressure; (3) organizational, related to care load and bio-cleansing; and (4) epidemiological, depending on whether the MDRO is endemic or epidemic, from which emanates the concept of colonization pressure.

Regarding the bacterial factors, multiple published studies have shown that environmental contamination is more frequent around patients colonized with bacteria of the genus *Klebsiella* spp. than around patients colonized with *Escherichia coli*. In fact, these studies suggest that *Klebsiella* spp., owing to its ability to form biofilms, can survive for a long time in the environment. This environmental contamination contributes to the intra-hospital spread of *Klebsiella* spp., which elucidates the reason behind their higher cross-transmission rates and high potential to cause outbreaks [28,29,30,31]. The survival of MDROs on surfaces increases the risk of transmission through the environment. The long survival duration of MDROs in the environment can create reservoirs, facilitating their transmission. The detection of environmental contamination in hospitals has mainly been studied for Gram-positive bacteria, with contamination rates of up to 64% and 94% around patients colonized with methicillin-resistant *Staphylococcus aureus* (MRSA) and VRE, respectively [32,33,34]. Among Gram-negative bacteria, the prevalence of environmental contamination by extended-spectrum β-lactamase producing *Enterobacterales* (ESBL-PE) has been reported to be between 4% and 19%, depending on the species [28,29]. Lerner et al., 2013 described environmental contamination around patients harboring CPE [32]. Thus, patients in direct contact with contaminated environments can effectively acquire MDRO. Indeed, various studies have shown that patients admitted to a room previously occupied by a patient colonized or infected with an MDRO, such as MRSA, VRE, or *Pseudomonas aeruginosa*, have an increased probability of developing colonization or infection with these pathogens [33,35,36,37].

In order to improve environmental bio-cleaning targeting and minimize the risks of transmission, one ought to first identify the patient-related risk factors, by virtue of which it is possible to pinpoint those hospitalized patients who are at high risk of dissemination. Patient-related factors are, by and large, the clinical backdrop that increases hospital environmental contamination, such as high degree of fecal dependence or incontinence and/or MDRO fecal abundance [38]. In point of fact, numerous studies have posited that a relationship exists between a patient’s clinical profile and the environmental contamination that transpires. One study revealed that patients colonized by MDRO and diarrheic contaminated their environment in 59% of cases [39]. Likewise, in another study the authors identified that, among the demographic and clinical studied characteristics, fecal incontinence was the only predictive factor that independently correlated with the environmental contamination of patients colonized with CPE [38]. By the same token, wounds infected with MDRO constitute a source of contamination (36% of cases), whether by way of direct physical contact or through fluid secretions [39]. In addition, research concerning patients at risk of environmental contamination has been approached in a myriad of ways. Several authors have attempted to identify individual risk factors related to environmental contamination. Hence, correlation between the relative abundance of VRE in stool and the percentage of positive environmental samples has been unequivocally documented. Congruently, individual colonization, defined by the number of positive sites, tallies with environmental contamination [40,41]. With respect to CPE, only one study has suggested a correlation between environmental contamination and relative fecal abundance. However, this was a one-day prevalence study and the authors did not determine individual risk factors to identify disseminator patients [32]. Additionally, other authors have advanced a correlation between rectal colonization rates and environmental contamination [40,42]. However, in a recent study, Lerner et al., 2015, evaluated the environmental dissemination of CPE by quantifying contamination in the vicinity of 34 colonized patients. Among 26 disseminator patients, the authors identified a distinct group of six (18%), dubbed the “superspreaders”, who were responsible for 79% of the environmental contamination detected. Moreover, these “superspreaders” were likely to have high rectal CPE concentrations. Therefore, the authors inferred that only 20% of colonized patients are, in fact, responsible for 80% of CPE’s environmental dissemination [38].

In light of all that has been presented, it is difficult to predict the risk of environmental contamination for a given patient. However, one can deduce the following trends from the literature data available to date: (1) a greater risk of environmental contamination, regardless of the bacterial species and resistance mechanisms, is to be considered around patients with fecal incontinence or diarrhea [38,39]; and (2) in the absence of a relevant clinical condition, significant environmental contamination is to be particularly considered for VRE, *Klebsiella* spp. and *Acinetobacter* spp. [28,29,30,31,33,36,43].

On the other hand, the transmission of MDROs from one hospitalized patient to another is possible via a vector, such as a healthcare professional. Morgan et al., 2012 observed that, out of 585 interactions between patients and caregivers, contamination of gloves and gowns was detected in 120 (20.5%) cases [17]. In a cohort study carried out around patients colonized with Gram-negative MDRO, the authors hypothesized that certain types of care, in particular, washing the perineum along with antibiotic consumption, were the factors associated with hand contamination in healthcare professionals. However, such contamination was rare, affecting only 7% of healthcare providers [44].

As the demonstration of direct transmission and acquisition of MRDOs through the environment is not experimentally feasible, the literature indicates that the necessary mechanisms for transmission do occur. However, to date most studies have been conducted during outbreaks and/or do not take into account confounding aspects related to individual risk factors for environmental dissemination [28]. One study has evaluated the risk factors associated with environmental contamination, correlating fecal abundance of MDROs with the risk of dissemination in a non-outbreak context. The authors argued that environmental contamination rates at hospitals are substantially higher for patients with VRE when compared to those with ESBL-PE and CPE. They found no difference in environmental dissemination between *Escherichia coli* and *Klebsiella pneumoniae* or between ESBL-PE and CPE, taking into consideration patients’ characteristics and individual risk factors for environmental dissemination, including Charlson’s score of comorbidities and Katz’s score of dependence [43].

Finally, an additional concept to consider when studying MDRO transmission dynamics is the prevalence of individuals colonized with the same MDRO in the same hospital ward at a given time, which is called “colonization pressure”. It is an important infection control metric that can be used to quantify the burden of an MDRO in a hospital unit, and can represent an estimate of the probability of cross-transmission within the unit [45]. A number of reports have uncovered an association between high colonization pressure and high transmission risk [46,47]. Bonten et al., 1998 sought to assess the effect of “colonization pressure” on VRE transmission in an intensive care unit in the Netherlands. The authors performed daily rectal screening throughout 19 weeks looking for VRE colonization in 181 consecutively admitted patients. They found that the risk of new acquisitions is multiplied by 3.2 for every 1% increase in the prevalence of patients colonized with VRE [46]. In parallel, Dalben et al., 2013, performed surveillance cultures in all patients on the third day of their admission to the intensive care unit of a hospital in Brazil and on a weekly basis during their stay, searching for colonization with carbapenem-resistant-*Pseudomonas aeruginosa* and multidrug-resistant-*Acinetobacter* spp. The authors found that for every 1% increase in colonization pressure by *Pseudomonas* spp. and *Acinetobacter* spp. there was a 2% increase in the incidence of cross-transmission [48]. For *Clostridioides difficile*, CPE, and MRSA, this relationship was found to be true in endemic situations [45,49,50,51,52]. A prevalence greater than 10% of patients colonized with CPE was an independent risk factor for new acquisitions among hospitalized patients. This prevalence was demonstrated by Torres-Gonzales et al., 2015, in a cohort study carried out in a hospital in Mexico during an outbreak of carbapenemase-producing *Klebsiella pneumoniae* type NDM-1 [51]. Other studies have evidenced that when VRE colonization pressure is greater than 50%, which is defined as a critical level, the risk of cross-transmission is sufficiently high that the effect of infection control measures, including compliance with hand hygiene, becomes insignificant [46,48]. Thus, in an endemic context monitoring the pressure of colonization with MDRO in specific care units constitutes an important means, although an indirect one, of controlling the risk of cross-transmissions and outbreaks [53].

## 3. Host Risk Factors for MDRO Acquisition

A healthy intestinal microbiota constitutes an important defense barrier against colonization by exogenous bacteria [54]. Acquisition and persistence of MDRO colonization is more common in dysbiosis. Alteration of the intestinal microbiota can be caused by antibiotic therapy, intestinal infections, and changes in diet [55]. For patients who are already colonized, the alteration of intestinal microbiota by antibiotic therapy promotes high-density colonization with an MDRO [40]. The intestinal microbiota is made up of 99% strict anaerobes, which were the subject of the very first studies in the 1970s. These studies have shown that the loss of anaerobic flora is correlated with a predisposition to infection or colonization with opportunistic bacteria [56,57]. *Enterobacterales* and enterococci represent a subdominant part of the healthy human gut microbiota. However, their proliferation is usually linked to alteration of the microbiota [58]. Intestinal microbiota can inhibit colonization with pathogenic or opportunistic bacteria by several mechanisms, including two main ones: a direct mechanism by competition and an indirect mechanism by modulating the host’s immune defenses [59]. The direct mechanism by competition involves type VI secretion system (T6SS), production of bacteriocin, competition for the acquisition of nutrients, and production of short-chain fatty acids, which is associated with the decrease in intestinal pH. The indirect inhibition mechanism includes the production of antimicrobial peptides and the stimulation of innate immunity by intestinal metabolites [60].

It is obvious that overconsumption of antibiotics at the human, animal, and environmental levels has aggravated the phenomenon of resistance. Indeed, the administration of an antibiotic has consequences on different microbiota: cutaneous, vaginal, respiratory, urinary, and mainly digestive. The latter seems to be the most impacted in part because of its richness and diversity [61,62,63]. Detection of MDROs when the intestinal microbiota is intact is problematic. In vitro gut modeling has shown that antibiotic exposure disrupts microbiota populations and allows CPE proliferation, increasing detection [64]. At an individual level, the selection pressure on intestinal microbiota caused by antibiotics favors colonization with MDROs. This may be the result of: (1) proliferation of bacteria with intrinsic or acquired resistance to the administered antibiotic, and (2) acquisition of exogenous pandemic MDROs [27,65].

## 4. International Policies to Limit the Spread of MDROs

Faced with the severity of the MDRO predicament and its individual and collective consequences, many scientific societies have developed recommendations to help healthcare teams control the spread of MDROs [30]. These international recommendations include a series of control measures, based essentially on a “search and isolate” policy, mainly including: surveillance cultures, the application of barrier measures ranging from patients’ isolation in single rooms with the reinforcement of hand hygiene, and the implementation of additional contact precautions such as the cohorting of colonized patients in a dedicated unit with or without a dedicated staff. In addition, most policies include the application of an antimicrobial stewardship program [66]. At the same time, in the event of a fortuitous discovery of a non-isolated colonized or infected patient, admissions and transfers in the corresponding ward are stopped and weekly screening is carried out over three weeks for all contact patients [67].

While these recommendations are accepted by all, they have not yet been proven effective outside of outbreak settings [68].

### 4.1. Surveillance Cultures

Conducting regular patient surveillance cultures allows for early identification of patients infected or colonized with an MDRO, which in turn expedites the isolation process, likely curtailing spread [69,70]. However, the success of this policy lies in the medial sector’s ability to recognize the populations at risk in order to better target their indications [71]. This endeavor can be challenging considering the specific epidemiological context of each country and the spread of MDROs in the community, which is insufficiently described in literature [72,73,74,75]. Surveillance is an extremely important tool to control the risk of MDRO dissemination in humans, animals, and the environment at several levels, including the community, subnational, national, regional, and global levels, according to the WHO One Health Approach [76].

In addition, the effectiveness of surveillance cultures is conditioned by the sensitivity of the various microbiological diagnostic techniques, their availability in certain countries, and their cost of use [77,78,79,80].

### 4.2. Isolation in Single-Bed Rooms

Isolating patients identified as colonized or infected with MDROs is a key measure to combat the risk of transmission between patients and members of the healthcare community [77]. Indeed, signaling a potential risk and delineating the associated geographical area can embolden healthcare workers and visitors to respect the indications for hand hygiene.

However, one of the main limitations of single-bed rooms is their availability. Moreover, the necessity of their use is currently debated. A recent publication argues that isolation in single-bed rooms for patients colonized or infected with ESBL-PE does not offer additional benefits over standard infection control policies [81]. The strategy of isolating patients in single-bed rooms has been criticized because of the suggested deleterious effects on the quality of care and the occurrence of adverse events. The main consequences of isolating patients are: (1) less supervision by healthcare workers (duration and number of visits), regardless of their status (medical or paramedical) [82]; (2) increased risk of preventable adverse effects, including dysglycemia, ventilator-associated lung disease, and errors in anticoagulant prescriptions [83]; (3) increase in the total duration of hospital care in regular medical units as well as in intensive care units [84]; and (4) occurrence of psychological consequences, including anxiety and depression [85].

### 4.3. Hand Hygiene

Hand hygiene is the most important measure in inhibiting the transmission of MDROs. Healthcare workers’ compliance with hand hygiene protocols is paramount [86,87]; a 100% compliance would prevent MDRO transmission through touch. Therefore, the level of compliance is important; in particular, scores of studies have shown that the observance of hand hygiene by healthcare workers remains insufficient. Su et al., 2015, stipulate that compliance before any intervention was only 51% in five Chinese intensive care units. After three months of rigorous training, compliance increased to only 67% [88]. In a prospective observational study to monitor hand hygiene compliance and the incidence of MDROs in clinical specimens, Trick et al., 2007, showed a significant decrease in the incidence of MDROs when hand hygiene compliance increased from 23 to 46%. However, in hospitals where the improvement rate was lower, no impact on the incidence of MDROs was observed [89]. In order to assess the impact of hand hygiene measures, Derde et al., 2014, performed a three-phase sequential study in thirteen intensive care units across eight European countries. Their study highlighted the importance of achieving over 70% compliance with hand hygiene in order to efficiently reduce the risk of MDRO transmission [90]. While hand hygiene compliance is only one aspect of the set of measures needed to combat the spread of MDRO, it remains the intervention with the greatest impact in mathematical simulation models [67,91].

### 4.4. Additional Contact Precautions

The effectiveness of standard precautions based primarily on hand hygiene is well established. However, they are generally insufficiently respected. Thus, the addition of contact precaution measures seems to be a rational way to limit the risk of cross-transmission of MDROs. This includes the use of gloves and gowns in contact with isolated patients and the use of dedicated care equipment when possible. One of the demonstrated benefits of additional contact precautions lies in improving the quality and compliance level of hand hygiene measures. In an observational study carried out in a hospital center in Mexico, Almaguer et al., 2013, demonstrated that hand hygiene compliance improved when using additional contact precautions (more than 90% vs. 68.9%) [92]. However, it is difficult to assess the real impact of additional contact precautions, as most studies have combined additional contact precautions with other interventions. Golan et al., 2006, recorded hand hygiene compliance in a 14-month crossover trial comparing compliance at two intensive care units during periods with and without a gown-use requirement. The authors found that the hypothesis that a gown-use requirement might improve hand hygiene compliance in the intensive care unit could not be confirmed. In the subgroup of patients with whom additional contact precautions were taken, the improvement in hand hygiene compliance associated with the gown-use requirement was small and did not affect pre-care rates [93]. Additional contact precautions for patients colonized with ESBL-PE identified through an extensive screening at a hospital level were not associated with a reduction in nosocomial transmission of the MDROs in question [94]. Similarly, other studies have shown that additional contact precautions have no impact on VRE cross-transmission [95,96]. In their three-phase sequential study, Derde et al., 2014, found no reduction in the acquisition rates of MRSA, VRE, and multi-drug resistant *Enterobacteria* after the introduction of additional contact precautions [90].

### 4.5. Cohorting

Cohorting is defined as grouping together those patients colonized or infected with the same MDRO in one care area in order to avoid contact with other susceptible patients [97]. The purpose of cohorting is to prevent cross-transmission from the hands of healthcare workers or from the environment [98]. In 1999, Austin et al. placed cohorting as the central method to stop cross-transmission of VRE in healthcare settings. Following the increase in VRE cases in the United States, the grouping of patients and healthcare workers has significantly reduced the number of secondary acquisition cases generated by a colonized index patient [99]. However, it was not until 2007 that Israel’s Minister of Health issued the first national guideline recommending the grouping of healthcare workers and patients to control an outbreak of carbapenemase-producing *Klebsiella pneumoniae*. An association was then observed between the increase in compliance with patient cohorting by dedicated staff and the reduction in the incidence rates of carbapenemase-producing *Klebsiella pneumoniae* [100]. In France, the implementation of a patient cohorting policy and dedicated staff within two days of admission of patients colonized or infected with CPE has made it possible to limit the number of people affected in outbreaks. However, in another study even quickly applying additional contact precautions was not sufficient to prevent cross-contamination [26]. A systematic review of the recent literature has shown that the majority of healthcare settings using cohorting policies have succeeded in reducing the incidence rate of CPE [101]. Cohorting has been integrated into infection prevention and control protocols in many countries, as shown by another literature review evaluating the effectiveness of control measures against the spread of CPE [67].

Thus, many studies have shown the effectiveness of cohorting in controlling outbreaks, whether intra-hospital [26] or at the national level [100]. However, there are no randomized controlled studies evaluating the role of cohorting in controlling the spread of MDROs outside of an outbreak setting. In addition, the application of patient cohorting policies with or without a dedicated staff is costly. Healthcare settings are not always able to implement this policy due to a lack of financial, physical, and human resources, particularly in countries with low to middle incomes. Indeed, these recommendations seem difficult to apply even in HICs, as suggested by a national survey conducted in France that highlighted the heterogeneous resources available from one French hospital to another [102].

### 4.6. Antimicrobial Stewardship Programs

Another salient recourse in controlling the spread of MDROs is ensuring the proper use of antibiotics. The implementation of comprehensive antimicrobial stewardship programs is recommended, as demonstrated by [30]. A recent systematic review and meta-analysis aiming to assess the various interventions for controlling the risk of transmission of multi-resistant *Enterobacterales* to hospitalized patients has suggested that the proper use of antibiotics is the only measure that considerably reduces colonization, in particular of those species belonging to *Escherichia coli* (*p* < 0.0001). In their review, 63 studies were included; the most commonly assessed interventions were antimicrobial stewardship policies and selective oral or digestive decolonization procedures [103]. In a three-phase sequential study by Derde et al., 2014, improved hand hygiene was associated with a reduction in the acquisition of MRSA, though not with VRE or multidrug-resistant *Enterobacterales* [90]. This could be explained by the fact that the measures adopted in their work to combat the spread of MDROs lacked policies for proper use of antibiotics. Arguably, the application of an efficient antimicrobial stewardship program necessitates an available multidisciplinary team and compliance with the application of established recommendations, which can be challenging in certain hospital settings.

## 5. Constraints on the Application of International Protocols in Low-to-Middle-Income Countries

The current epicenter of resistance is stationed in LMICs. However, data on clinical resistance in Gram-negative bacilli, especially in *Enterobacterales*, are limited in these countries [104,105].

The international recommendations to control the spread of MDROs are based on the assumption that most colonized patients are at high risk of contaminating the hands of healthcare professionals as well as the immediate environment. They fall back on the idea that isolation in a single room, additional contact precautions, or even less standard precautions may not prevent MDRO transmission. In this context, the only other approach is cohorting. Nevertheless, cohorting, along with microbiological monitoring, is difficult to implement and requires supplementary human and physical resources. These resources are mostly in shortage in LMICs. In addition, the cessation of activities (admissions and transfers of colonized or infected patients and contact patients in the event of a fortuitous discovery) can lead to a considerable reduction in care level and an increase in costs [106].

A multicenter study including three French hospitals gauged the cost effectiveness of cohorting. Direct and indirect costs related to the additional human resources, microbiological diagnosis techniques, and interruption of new admissions were calculated. The average cost associated with the management of a single case identified upon admission and within the first 48 h post-admission was EUR 4443 ± 11,552 and EUR 11,445 ± 15,743, respectively (*p* < 0.01). The additional cost linked to an outbreak episode varied from EUR 14,864 ± 17,734 for an outbreak with a single secondary case to EUR 136,525 ± 151,231 when an outbreak generated several secondary cases. In the case of a colonized patient discovered on admission and isolated, the application of additional contact precautions and microbiological screening techniques represented 51% and 30% of the overall cost, respectively. In the event of an outbreak, costs related to the interruption of new admissions represented 77–94% of the total cost, and had the largest financial impact [106]. Furthermore, an assessment of the average cost associated with a CPE outbreak in five hospitals in the United Kingdom was reported at approximately EUR 1.1 million over ten months [107].

Finally, an increase in the prevalence of colonization with MDROs in the general population [108] and an increase in their incidence in hospital environments [109] has been found to engender a large resistance reservoir. Sequentially, this translates into difficulty in keeping all colonized patients in a single room and in contact tracing over a three-week period, along with adverse events potentially related to patient isolation [85].

For LMICs, this quandary is much more complex. The combination of high levels of antibiotic use in humans, animals, and agriculture along with poor infrastructure (inadequate sewage systems, poor quality drinking water, and overcrowding) accelerates the spread of MDROs within the general population [110]. In fact, the practice of self-diagnosis and self-medication with antibiotics by patients themselves and restraint with respect to pharmacist advice is widespread in LMICs [111].

In addition, most of said countries lack the microbiological expertise and resources to cultivate and identify MDROs. The realistic likelihood of implementing the aforesaid international policies to control the spread of MDROs in these countries is slight [112]. Indeed, the limited human and material resources of the majority of the healthcare establishments in LMICs make the application of additional contact precautions unattainable. Moreover, the costs associated with the use of over-gowns and gloves are sometimes prohibitive [53]. Likewise, an international survey of epidemiologists from different hospitals in LMICs found that the presence of single rooms was infrequent. The absence of administrative support and national recommendations are real impediments to the application of international policies. Therefore, a tailor-made policy and rational employment of resources have to substitute for international policies [53,113].

## 6. Possible Targeted Strategies Adapted to the Means of Low-to-Middle Income Countries

Recent publications have suggested that contact isolation precautions and/or single-bed rooms for patients colonized or infected with ESBL-PE do not offer additional benefits over standard infection control precautions [81,114]. Indeed, a cluster randomized non-inferiority study showed that an isolation strategy of contact precautions in multi-bed rooms was not inferior to a strategy of contact precautions in single-bed rooms for controlling the risk of cross-transmission [81]. This study was conducted in the medical and surgical departments of 16 hospitals in the Netherlands, and aimed to compare patients colonized with ESBL-PE isolated in individual rooms with others admitted in multi-bed rooms. More recently, a cross-cluster randomized study in 20 medical and surgical departments of four European university hospitals compared additional contact precautions to standard precautions in preventing ESBL-PE acquisition. Patients hospitalized for at least one week were screened with rectal culture within three days of admission, once a week, and at discharge. The primary outcome was incidence density of ESBL-PE, defined by the acquisition rate per 1000 patient-days. The authors showed that additional contact precautions had no additional advantage compared to standard precautions [114]. Because ESBL-PE transmission shares several aspects in common with CPE transmission, including resistance plasmid vector and spread within commensal species, it is possible to extrapolate that the application of additional contact isolation precautions with CPE-colonized or infected patients would generate no added value over standard precautions in controlling the risk of hospital dissemination.

Strongly held opinions about the need for contact isolation to prevent the spread of MDROs in hospital settings have contributed to increased costs and decreased flexibility, and have driven aggressive diagnostic testing for colonization in asymptomatic patients. Examination of the evidence cited in support of the benefits of isolation and growing evidence of its unintended harms offer an opportunity to reevaluate how contact isolation might best be applied. Indeed, in a mini-literature review, Kirkland highlighted that, when considering the real-world settings in which clinicians practice, it is likely that the benefit-to-harm balance of contact isolation varies according to different circumstances. This implies that an individualized approach is justified. For example, in the case of a healthy patient admitted electively to a hospital with a clean and spacious environment, a high level of hand-hygiene compliance, and low rates of hospital-acquired infections, the benefits of contact isolation may be outweighed by the harms. However, in the case of a patient with a large draining wound in a crowded hospital with a low level of hand-hygiene compliance and an epidemic level of MRSA infection, contact isolation may be legitimate [115].

In order to assess the impact of hand hygiene measures associated with chlorhexidine body-washing and the isolation of patients colonized with MRSA, VRE, or multi-resistant *Enterobacterales* on preventing the risk of acquisition, Derde et al., 2014, performed a three-phased sequential study in thirteen intensive care units across eight European countries. The first observational phase lasted six months, and acted as the control period associated with data collection at baseline. The second interventional phase lasted over six months, and consisted of reinforcing compliance with hand hygiene, associating training programs, audits, and a daily skin decontamination policy using chlorhexidine body-washing applied to all patients. The third interventional phase lasted 12 months, and included the implementation of a screening policy and additional contact precautions for patients identified as colonized or infected by an MDRO. The key takeaways were embodied in a significant reduction in acquisition rates of MRSA in the second intervention period following improved hand hygiene combined with universal chlorhexidine body-washing (observance of hand hygiene went from 52% in period 1 to 69% in period 2, then to 77% in period 3). The same intervention did not reduce acquisition of VRE and multi-resistant *Enterobacterales*. Implementation of screening policies and contact isolation for carriers had no incremental effect on MDRO acquisition. This study sheds light on the stipulation that in the context of a sustained high level of compliance with hand hygiene (over 70%) and chlorhexidine body-washing, the screening and isolation of carriers does not reduce MDRO acquisition rates [90].

Alternatively, Harris et al. 2021 demonstrated in a cluster randomized study that universal glove and gown use in intensive care units was associated with a non-statistically significant decrease in acquisition of multidrug-resistant Gram-negative bacilli [116].

Moreover, in a cohort study of patients colonized with multidrug-resistant Gram-negative bacilli, the authors suggested that certain types of care, in particular washing the perineum and the prescription of antibiotics, were the only factors associated with contamination of the hands in healthcare professionals. However, such cross-contamination was rare; only 7% of the healthcare workers had their hands contaminated [44]. Thus, the data from the literature shows that transmission of Gram-negative bacilli within hospital structures depends on compliance with standard precautions, and probably includes the importance of the source itself.

A multifaceted strategy composed of contact precautions, antimicrobial stewardship programs, environmental cleaning, and chlorhexidine body-washing was the most effective intervention to prevent multidrug-resistant Gram-negative bacilli acquisition in the overall analysis and for each type of bacteria. This was demonstrated by a systematic review and a network meta-analysis which aimed to evaluate the relative efficacy of different strategies for the prevention of transmission of multidrug-resistant Gram-negative bacilli in adult intensive care units. However, in the subgroup of multi-drug resistant *Acinetobacter baumannii*, three-component strategies containing environmental cleaning and/or chlorhexidine body-washing as a core component were associated with reduced acquisition. In the subgroup of ESBL-PE strategies, those with antimicrobial stewardship programs as a core component were strongly associated with a decrease in acquisition. The effect of environmental cleaning on the prevention of multidrug-resistant Gram-negative bacilli acquisition and infection was significant only among studies conducted in European countries. The authors found a relationship between the number of interventions and outcome (i.e., colonisation and infection). This suggests that when opting for effective interventions, infrastructure and resource availability should be considered first and foremost [117].

Another systematic review and reanalysis of quasi-experimental studies showed that multimodal infection prevention and control strategies including at least three integrated components are essential to significantly prevent the risk of multidrug-resistant Gram-negative bacilli transmission. Indeed, multimodal infection prevention and control strategies with specific components are critical in the context of local epidemiology and resource scarcity [101].

These data suggest that the adoption of an alternative policy is conceivable. Individual hospitals can contemplate interventions based on the importance of different MDROs, their burden on different units, their significance, and the cost of intervening against them [116]. In Table 1, we propose adapted policies to limit the spread of MDROs while taking into account the bacterial species and those hospital wards considered at highest risk.

**Figure 1:**
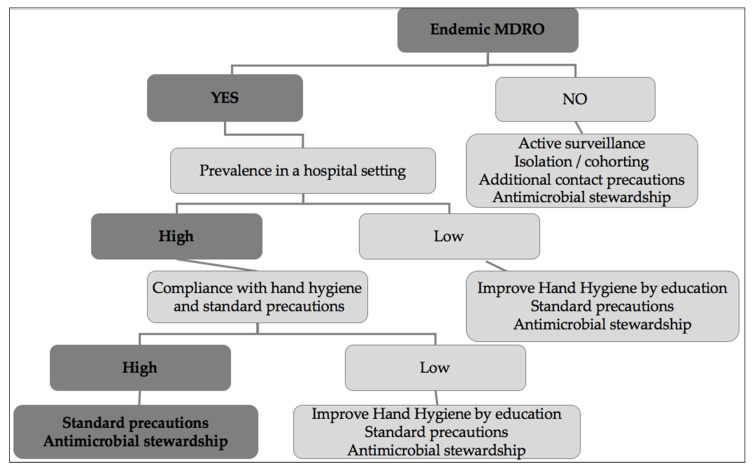
Proposed targeted strategy to limit the spread of MDROs.

We propose targeted strategies in hospital settings adapted to the epidemiological characteristics of different MDROs based on whether the MDRO is endemic or not (Figure 1). In LMICs, in a setting where a MDRO is endemic and its prevalence is high, high compliance with hand hygiene and effective antimicrobial stewardship program could be sufficient to limit the risk of transmission. However, if compliance with hand hygiene is low, contact precautions must be applied in addition to education programs aiming to improve hand hygiene application. In a context of endemic MDROs with low prevalence in the setting, effective hand hygiene measures and antimicrobial stewardship can be sufficient to prevent the risk of transmission if continuous education programs are arranged focusing on improving hand hygiene. As for HICs where MDROs are not endemic, the application of international infection-prevention policies relying on screening, isolation, and cohorting strategies for colonized patients should be considered.

As a result, it may be possible to discontinue contact isolation for colonized or infected patients with MDROs in hospital settings, considering MDRO’s incidence, predominant species, and regional prevalence together with the level of compliance with hand hygiene and the application of an appropriate antimicrobial stewardship program (Table 2).

## 7. Conclusions

There is sufficient evidence to indicate that high compliance with hand hygiene and antimicrobial stewardship reduce the risk of MDRO transmission, though without entirely eliminating it. In LMICs, it would be possible to adopt a policy relying solely on these two factors without necessarily having to implement additional costly isolation and contact precautions. Compliance with hand hygiene, however, must exceed 70%. Nonetheless, a category of patients who are considered superspreaders due to being colonized or infected with particular bacterial species or with a specific clinical profile require isolation. The major limitation of our review is the fact that it is based on studies conducted mainly in HICs. Considering the impracticality of international standards in resource-stricken countries, it is necessary to consider and test targeted strategies to effectively fend off the further escalation of antimicrobial resistance.

## Figures and Tables

**Table 1:** Variables to take into account when choosing and adapting a policy to limit the spread of MDROs in hospital settings.

		High Compliance with Hand Hygiene	Antimicrobial Stewardship	Active Surveillance	Patient Isolation	Additional Contact Precautions	Cohorting
Multi-drug resistant bacterial species	*Escherichia coli*	✓	✓	×	×	×	×
	Other *Enterobacterales*	✓	✓	×	×	×	×
	VRE	✓	✓	✓	✓	✓	✓
	*Pseudomonas aeruginosa* producing carbapenemase	✓	✓	×	×	×	×
	*Acinetobacter baumannii* resistant to carbapenems	✓	✓	✓	✓	✓	✓
Hospital wards	Long term care facilities	✓	✓	×	×	×	×
	Intensive care units and reanimation	✓	✓	✓	✓	✓	✓*
	Other wards	✓	✓	×	×	×	×
Patient clinical characteristics	Diarrhea and or fecal incontinence	✓	✓	✓	✓	✓	✓
	High Katz score	✓	✓	✓	✓	✓	✓
	Other patients	✓	✓	×	×	×	×

✓: To adopt. ✓*: To adopt in an ongoing outbreak context. ×: Not to adopt. VRE: Vancomycin-resistant enterococci.

**Table 2:** Variables to consider for discontinuing contact isolation in LMICs.

Variables	Target
Incidence of MDRO	<national median incidence
Predominant species	*E* *scherichia* *coli*
Compliance with hand hygiene	>70%
Regional prevalence	low
Antimicrobial stewardship program	Applied
